# A Novel Thylakoid Ascorbate Peroxidase from *Jatrophacurcas* Enhances Salt Tolerance in Transgenic Tobacco

**DOI:** 10.3390/ijms15010171

**Published:** 2013-12-24

**Authors:** Zhibin Liu, Han Bao, Jin Cai, Jun Han, Lirong Zhou

**Affiliations:** 1 Key Laboratory of Bio-Resources and Eco-Environment of Ministry of Education, College of Life Sciences, Sichuan University, Chengdu 610064, Sichuan, China; E-Mails: liuzhibin@scu.edu.cn (Z.L.); baohan163@163.com (H.B.); cassandra.cai@gmail.com (J.H.); 2 West China School of Pharmacy, Sichuan University, Chengdu 610064, Sichuan, China; E-Mail: shiyan805@126.com; 3 Architecture & Environment Department, Sichuan University, Chengdu 610065, Sichuan, China; 4 Department of Civil Engineering, University of British Columbia, Vancouver, BC V6T 1Z4, Canada

**Keywords:** abiotic stress tolerance, anti-oxidative enzymes, ascorbate peroxidase, salt stress, *Jatropha curcas*

## Abstract

Ascorbate peroxidase (APX) plays an important role in the metabolism of hydrogen peroxide in higher plants. In the present study, a novel *APX* gene (*JctAPX*) was cloned from *Jatropha curcas* L. The deduced amino acid sequence was similar to that of APX of some other plant species. JctAPX has a chloroplast transit peptide and was localized to the chloroplasts by analysis with a JctAPX-green fluorescent protein (GFP) fusion protein. Quantitative polymerase chain reaction (qPCR) analysis showed that *JctAPX* was constitutively expressed in different tissues from *J. curcas* and was upregulated by NaCl stress. To characterize its function in salt tolerance, the construct *p35S: JctAPX* was created and successfully introduced into tobacco by *Agrobacterium*-mediated transformation. Compared with wild type (WT), the transgenic plants exhibited no morphological abnormalities in the no-stress condition. However, under 200 mM NaCl treatment, *JctAPX* over-expressing plants showed increased tolerance to salt during seedling establishment and growth. In addition, the transgenic lines showed higher chlorophyll content and APX activity, which resulted in lower H_2_O_2_ content than WT when subjected to 400 mM NaCl stress. These results suggest that the increased APX activity in the chloroplasts from transformed plants increased salt tolerance by enhancing reactive oxygen species (ROS)-scavenging capacity under short-term NaCl stress conditions.

## 1. Introduction

In recent years, salinity has become a major abiotic stress that severely affects plant growth and agricultural productivity. Salt stress may induce alterations in biochemical pathways and physiological responses [1]. Under salt stress, both ionic and osmotic balances of plants are perturbed, thereby inducing an increase of reactive oxygen species (ROS) [2], including hydrogen peroxide (H_2_O_2_), superoxide anions (O_2_•^−^), hydroxyl radicals (•OH) and singlet oxygen (^1^O_2_). The accumulation of high levels of ROS results in perturbing or overwhelming anti-oxidative defenses, which could lead to severe damage because high ROS affect the integrity of cellular membranes and the activity of various enzymes [3], reduce nutrient uptake, and alter photosynthesis [4].

Because oxidative stress is an important component of salt stress [5], efficient detoxification of ROS is an important component of salt tolerance [6,7]. Plants have an array of enzymes in different cell organelles that work in concert to scavenge ROS. A major hydrogen peroxide detoxifying system in plant chloroplasts and cytoplasm is the ascorbate-glutathione cycle, in which ascorbate peroxidase (APX; EC 1.11.1.11) acts as the key enzyme [8]. This enzyme uses ascorbate as an electron donor to reduce H_2_O_2_ to water. APX has been identified in many higher plants, with different isozymes distributed in at least four cellular compartments, including the cytosol, peroxisomes, mitochondria and chloroplasts [9,10]. Chloroplast APX includes two isoforms, one in the stroma and the other associated with the thylakoid membranes. Both isoforms play important roles in photosynthesis when plants are stressed.

*Jatropha curcas*, commonly known as physic nut, belongs to the family Euphorbiaceae and is abundantly distributed in many tropical and sub-tropical regions throughout the Americas, Africa, and Asia [11]. The plant is characterized by its hardiness, ease of propagation, endurance during drought, high oil content, low seed cost, short gestation period, rapid growth, adaptation to a wide range of agro-climatic conditions, and a bushy/shrub-like nature [12]. Different parts of *J. curcas* have been used for various purposes, such as medicine production, animal feeding and cosmetic production [13]. Recently, *J. curcas* has received a lot of attention as a potential source of renewable energy from its relatively oily (27%–40%) seeds, which are easily converted into biodiesel that meets American and European standards [14]. This species has drought, salinity, and pest resistance, enabling it to grow in areas that are not suitable for most other agriculturally important plants. Previous studies have shown that the antioxidant response to oxidative stress might be one of the most important factors of the tolerance of *J. curcas* against abiotic stress conditions [15]. However, in contrast to other plants, the key enzymes of *J. curcas* have not been well characterized at the molecular level.

In the present study, a novel *JctAPX* gene was cloned from *J. curcas*. We detected the expression of *JctAPX* in different tissues of *J. curcas* and when stressed with salt. Subcelluar localization of JctAPX was analyzed by using a green fluorescent protein (GFP) fusion protein. To characterize the role of JctAPX *in vivo*, it was overexpressed in tobacco *Nicotiana tabacum*. The differences between transgenic and wild type (WT) plants were compared under NaCl stress.

## 2. Results

### 2.1. Cloning and Characterization of the *JctAPX* Gene

**Figure 1.**
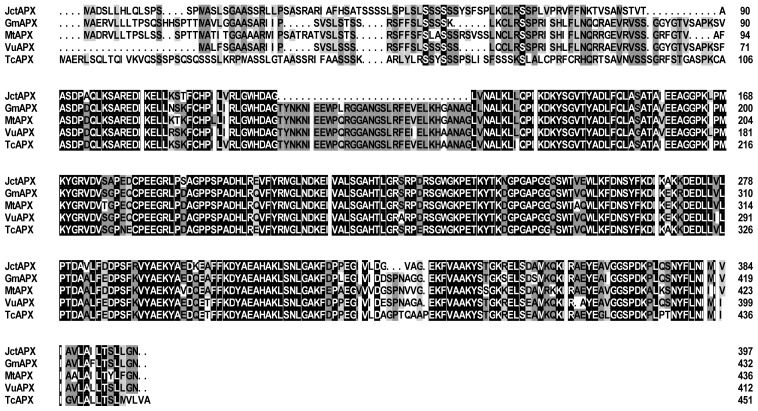
Amino acid sequence alignment of APXs from several plant species using the software Clustal X. The GenBank accession numbers and names for these sequences are as follows: JctAPX, *J. curcas* tAPX (KF560614); GmAPX, *Glycine max* APX (XP 003526640.1); MtAPX, *Medicago truncatula* (XP 00360245.1); VuAPX, *Vigna unguiculate* (AAS55852.1), and TcAPX, *Theobroma cacao* (EOY 16335.1). Identical and similar amino acid residues are shaded black, and dashes indicate gaps introduced to optimize alignment.

**Figure 2.**
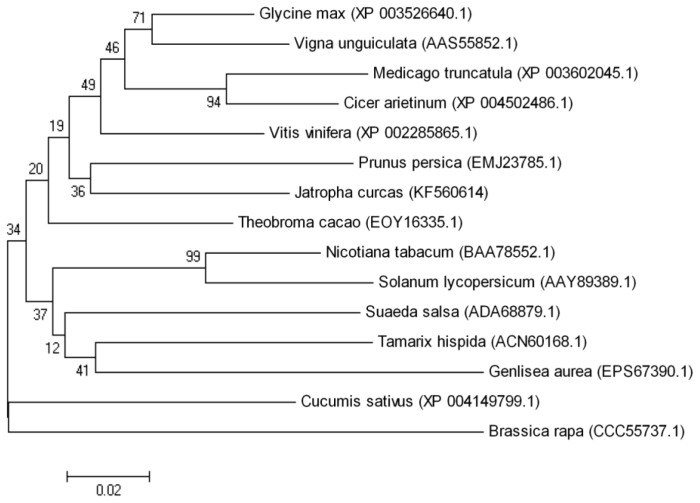
Phylogenetic tree showing the respective affiliations of various APX proteins from higher plants. The sequences were obtained from GenBank and aligned with that of JctAPX. The GenBank accession numbers are given in parentheses. The tree was constructed using the MEGA4 software.

*tAPX*s have been cloned from many plants, and sequence analysis has revealed a conserved region [16], which was used to design the degenerate primers to clone the *tAPX* gene from *J. curcas*. A 327-bp fragment was amplified from the cDNA of *J. curcas* leaves. The full-length cDNA, named *JctAPX* (GenBank accession number: KF560416), was obtained by 5′ and 3′-rapid amplification of cDNA end (RACE). The cloned *JctAPX* gene consisted of 1194 base pairs that encoded a polypeptide of 397 amino acid residues with a calculated molecular mass of 42.84 kDa. Sequence alignment of the deduced amino acid sequence (Figure 1) showed that it was approximately 70% identical to its homologues in *Glycine max*, *Medicago truncatula*, *Vigna unguiculata*, and *Theobroma cacao*. Highly conserved amino acid sequences appeared in the *C*-terminal regions, whereas non-conserved sequences existed in the *N*-terminal regions (Figure 1). In addition, phylogenetic analysis based on a neighbor-joining (NJ) bootstrap method indicated that JcAPX was most closely related to PpAPX from *Prunus persica* (Figure 2).

### 2.2. Subcellular Localization of JctAPX

**Figure 3.**
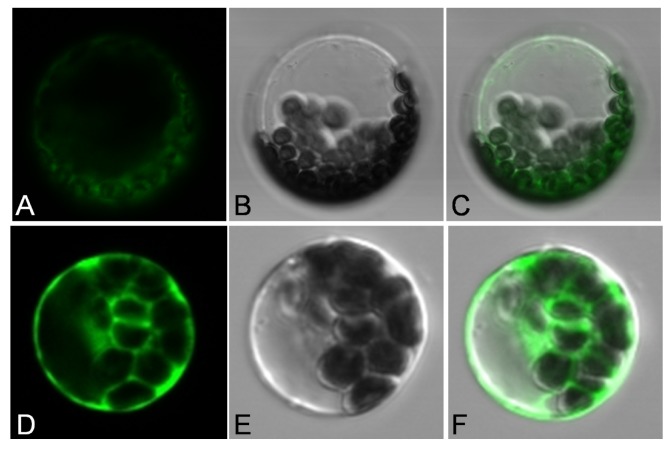
Intracellular targeting of JctAPX in *Arabidopsis. thaliana* protoplasts. (**A** & **D**) Green fluorescence of JctAPX-GFP and GFP fusion protein, respectively; (**B** & **E**) Images of protoplasts in bright fields; and (**C** & **F**) Merged images of **A**, **B** and **D**, **E**, respectively.

TargetP software predicted the chloroplast localization of JctAPX and a chloroplast transit peptide of approximately 83 amino acids. Subcellular localization of JctAPX was confirmed by GFP fluorescence. We performed targeting experiments *in vivo* in *Arabidopsis* protoplasts derived from leaf tissue. In the protoplasts transfected with *p35S: JctAPX-GFP*, which expressed the JctAPX-GFP fusion protein, the green fluorescence was clearly associated with chloroplasts and co-localized with the chloroplasts. By contrast, in the protoplasts transfected with the control construct *p35S: GFP*, the green fluorescence was distributed in the cytoplasm surrounding the chloroplasts and was not co-localized with the chloroplasts (Figure 3).

### 2.3. Comparison of Expression Levels of *JctAPX*

**Figure 4.**
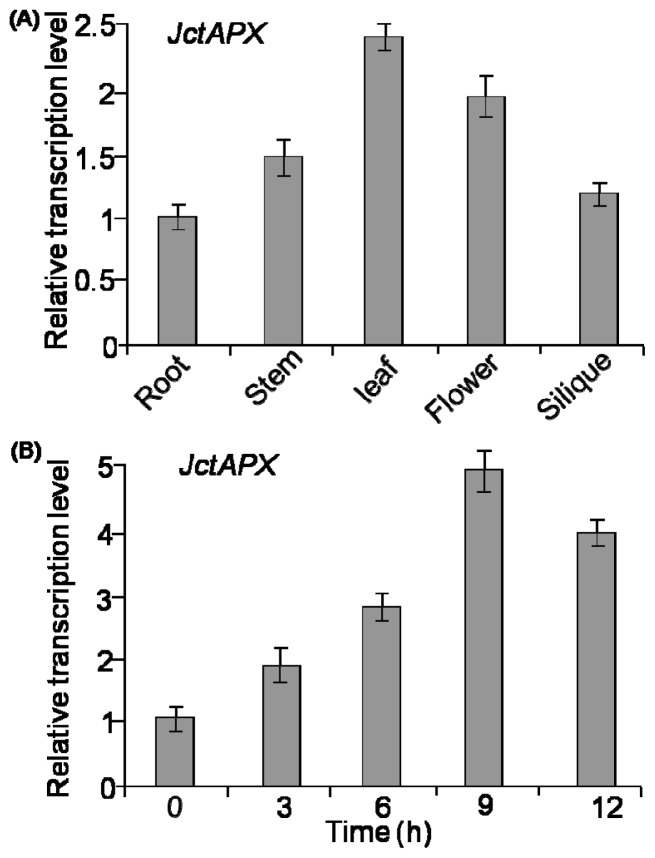
(**A**) The expression of *JctAPX* in different *J. curcas* tissues. The root, stem, leaf, flower, and silique were harvested, and total RNAs were extracted to run qRT-PCR. The amount of root mRNA expression was set as 1 for reference. The data are means of four separate runs and standard deviations (SDs) are indicated; and (**B**) The level of *JctAPX* transcripts induced by NaCl stress. Total RNAs were isolated from 4-week-old leaves of *J. curcas* treated with 400 mM NaCl, and then analyzed by qRT-PCR. The transcripts of *actin* were used as a control, and the amount of WT mRNA expression at 0 h was set as 1. The data are means of four separate runs and SDs are indicated.

The expression of *JctAPX* in different tissues was analyzed in order to determine its spatial expression pattern. The abundance of the *JctAPX* gene in different tissues was measured by quantitative reverse transcription-polymerase chain reaction (qRT-PCR). The results indicated that the *JctAPX* gene was expressed in all *J. curcas* tissues (the root, stem, leaf, flowers and silique). The expression of *JctAPX* was significantly higher in the leaf tissue compared to other tissues (Figure 4A).

To investigate the possible function of *JctAPX* in response to salt stress, we analyzed its expression level in the presence of 400 mM NaCl. The results showed that *JctAPX*’s expression was remarkably up-regulated under NaCl stress in a time-dependent manner. After a 3 h salt treatment, the expression level of *JctAPX* was increased up to 1.9 times that of the control, and reached a peak after 9 h of treatment (Figure 4B). These results indicated that *JctAPX*’s function might be related to the plant’s salt response.

### 2.4. Molecular Characterization of Transgenic Tobacco

**Figure 5.**
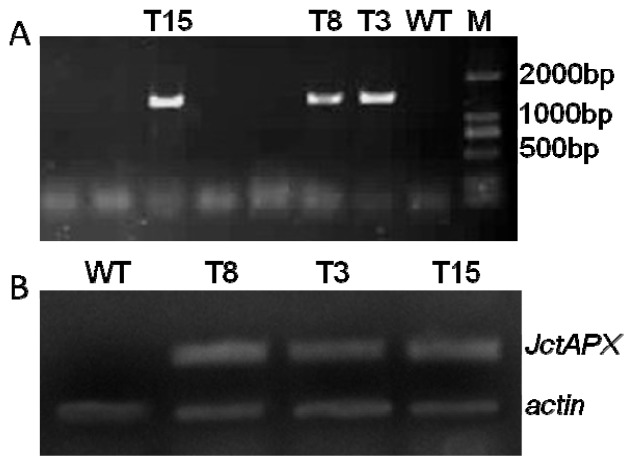
Molecular identification of tobacco plants transformed with *JctAPX*. (**A**) PCR identification of tobacco plants transformed with *JctAPX*. Lane M, DNA marker (DL2000); and (**B**) Over-expression of *JctAPX* in transgenic lines was confirmed by semi-quantitative RT-PCR. *Actin* was used as a reference to show that equal amounts of RNA were used in the analysis. WT, wild type; T3, T8 and T15, independent transgenic tobacco lines.

The fact that *JctAPX* expression was responsive to NaCl stress prompted us to analyze its function in NaCl-stress resistance. Accordingly, the *p35S: JctAPX* construct was introduced into *tobacco* plants by *Agrobacterium tumefaciens*. Transgenic plants were detected by PCR after the first screening with 50 μg/mL kanamycin. The upstream primer of pBI121 and the 3′-primer of the *JctAPX* gene were used in the amplification, and an intense 1300 bp band corresponding in size to the *JctAPX* product was obtained from some kanamycin-resistant plants, whereas no bands were produced from WT plants (Figure 5A). There were 10 individual transgenic lines harvested. Subsequently, the *JctAPX* levels in these transgenic plants were analyzed by semi-quantitative RT-PCR. The results showed that seven of the ten plants had strong positive signals, while no signal was found in the WT plants. Three transgenic lines (T3, T8, and T15) that expressed relatively higher levels were used for further analysis (Figure 5B).

### 2.5. Salt Tolerance in Transgenic Tobacco Overexpressing *JctAPX*

**Figure 6.**
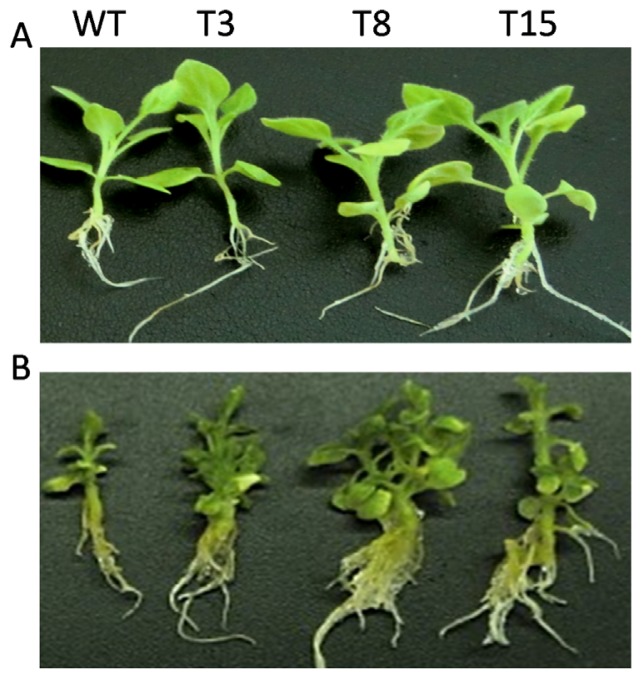
Growth of WT and transgenic tobacco seedlings on MS medium supplemented with 200 mM NaCl and untreated. Seven-day-old seedlings were transferred to MS agar plates supplemented with 200 mM NaCl or un-supplemented, and allowed to grow for four weeks. (**A**) Normal condition; and (**B**) 200 mM NaCl treatment. WT, wild type; T3, T8 and T15, independent transgenic tobacco lines.

WT and transgenic lines were treated with different concentrations of NaCl. As shown in Figure 6A, the transgenic plants grew as well as WT plants under normal conditions (no NaCl added) in Murashige and Skoog (MS) medium. When 200 mM NaCl was added, the growth of both WT and transgenic lines was inhibited. However, transgenic plants displayed better growth when compared to WT after a 30 days period. During this period, the leaves of WT plants gradually lost their greenness and root elongation was severely delayed, whereas leaves of the transgenic plants remained green and the roots displayed tolerance against salt stress (Figure 6B).

**Figure 7.**
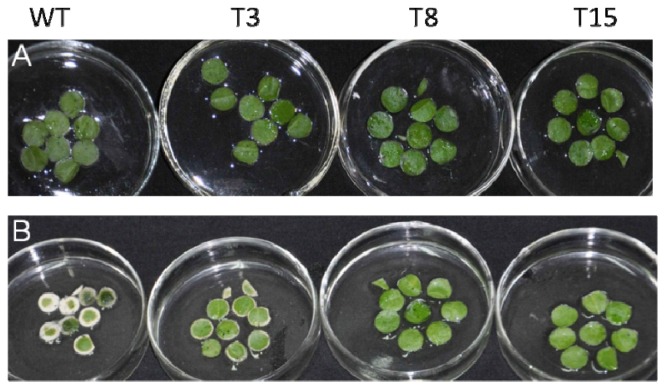
Visible damage to tobacco leaf discs exposed to salt treatment for 4 days. (**A**) Normal condition; and (**B**) 400 mM NaCl treated. WT, wild type; T3, T8 and T15, independent transgenic tobacco lines.

Leaf discs of transgenic and WT tobacco were then floated in MS solution containing 400 mM NaCl for 4 days, and the plant salt tolerances were examined by comparing phenotypes and chlorophyll contents. After 4 days of salt treatment, leaf discs from WT plants were bleached, whereas leaf discs from transgenic *JctAPX* plants remained green (Figure 7A,B).

**Figure 8.**
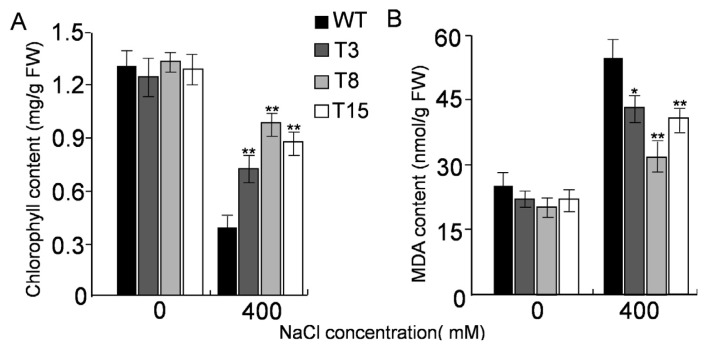
Effects of salt treatment on WT and transgenic tobacco lines. (**A**) Spectrophotometric quantification of chlorophyll contents of leaf discs treated with different concentrations of NaCl; and (**B**) MDA content analysis in transgenic tobacco lines and WT under salt stress. WT, wild type; T3, T8, and T15, transgenic lines. Values are expressed as means (*n* = 3); errors bars show the SD for each experiment. The single (*) and double asterisks (**) represent significant differences as determined by the Student’s *t*-test at *p* < 0.05, and *p* < 0.01, respectively.

Chlorophyll content measurements in these plants confirmed the observed phenotypic differences. The chlorophyll content in transgenic lines was noticeably higher than that in WT plants. Among the transgenic lines, line T8 had the highest chlorophyll content compared to the T3 and T15 transgenic lines (Figure 8A).

By contrast, all of the transgenic seedlings had lower malondialdehyde (MDA) content than WT seedlings both in the presence and in the absence of NaCl stress. Analysis of the variance showed that the MDA content in WT and transgenics lines was noticeably different under normal conditions, and significantly more different when under NaCl stress (Figure 8B).

These results suggest that over-expression of *JctAPX* can enhance tolerance for salt stress in transgenic tobacco.

### 2.6. Activities of tAPX and H_2_O_2_ Level under Salt Stress

**Figure 9.**
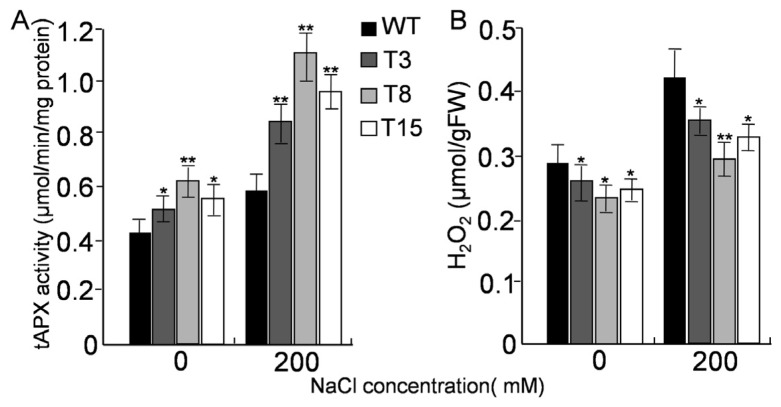
Changes in tAPX activity (**A**) and H_2_O_2_ content (**B**) in WT and transgenic plants under salt stress. Plants were treated with 200 mM NaCl for 24 h and sampled for measurement. Data points represent the mean values ± standard deviation (M ± SD) of three replications. The single (*) and double asterisks (**) represent significant differences determined by the Student’s *t*-test at *p* < 0.05, and *p* < 0.01, respectively.

It was hypothesized that the increased tolerance to salt stress might be due to the increase in tAPX levels. Indeed, the tAPX activity was higher in the transformed lines than in WT plants. When plants were exposed to 200 mM NaCl, tAPX activities in both WT and transgenic lines increased remarkably. After 24 h of treatment, the tAPX activity increased up to 143%, 168%, 185% and 171% in WT, T3, T8, and T15, respectively (Figure 9A).

Since the main function of APX is scavenging H_2_O_2_, it is necessary to detect endogenous H_2_O_2_ in transgenic and WT plants. The H_2_O_2_ content of plant leaves was quantified. Under normal growth conditions, H_2_O_2_ accumulation was low. After treatment with 200 mM NaCl for 24 h, WT plants accumulated more H_2_O_2_ than transgenic plants. Lines T3, T8, and T15 exhibited approximately 15%, 25%, and 21% lower H_2_O_2_ concentration, respectively, than WT (Figure 9B).

## 3. Discussion

APXs are important proteins reported to be involved in the response to different environmental stresses such as drought, low/high temperature, salinity, and high light intensity [17]. In this study, a novel *APX* gene was isolated from *J. curcas*. Sequence alignment of JctAPX with other plants suggests that *JctAPX* likely has almost the same function as other reported homologous proteins. Our present research showed that the *JctAPX* gene was expressed in all *J. curcas* tissues examined, and that it was gradually and strongly induced by NaCl stress (Figure 4A,B). Large increases in transcription levels of *APX* genes were also observed in *Vigna unguiculata* when subjected to progressive drought [18], and similar results of *StAPX* induced by salt and osmotic stresses were found in tomato leaves [19]. By contrast, constitutive expression of tAPX activity was observed and no significant changes in tAPX activity were found in other experiments [20,21]. Genomic and cDNA of *APX* sequences obtained from a wide variety of plant species have shown that APX is widely distributed in the plant kingdom. These enzymes are found in several cellular compartments, such as the cytosol, mitochondria, and chloroplast. In this study, the subcellular localization analysis indicated that JctAPX was targeted to chloroplasts, which is the compartment associated with the high-energy photosynthetic electron transport system and a generous supply of oxygen, making it very prone to ROS damage [8]. APX enzymes, especially those involved in the chlAPX pathway, constitute an important mechanism that protects plants from damage caused by H_2_O_2_ resulting from salt stress in this organelle [20,22].

Increasing evidences indicates that the activities of antioxidative enzymes are involved in tolerance to abiotic stresses [23–26]. Since transcription of the *JctAPX* gene was strongly upregulated by 400 mM NaCl, we hypothesized that it might play an important role in plants coping with salt stress. The role of *JctAPX* was demonstrated further by using a transgenic strategy. Under non-stress conditions, growth and development were similar for overexpressing transgenic lines and WT, which implied that *JctAPX* might not play a significant role under normal growing conditions. These results agree with those observed for *Populus* peroxisomal *APX* (*PpAPX*) overexpression lines, which did not display any major abnormalities in growth and development under normal growing conditions [27]. Although germination, cotyledon growth, and survival of all lines were influenced by salt stress, the transgenic lines showed increased tolerance to NaCl compared to the WT. Furthermore, no significant difference was found in the chlorophyll content among the tested lines when NaCl was absent. The chlorophyll content was higher, however, in WT than in transgenic tobacco at 400 mM NaCl. Whether under NaCl stress or not, APX activity was much higher in transgenic lines than in WT, which resulted in a lower H_2_O_2_ level in the transgenic lines than in WT. The metabolic balance of cells was likely disrupted by NaCl stress, resulting in enhanced production of ROS. Detoxification of ROS is linked to maintenance of the Calvin cycle in chloroplasts [28]. The high level of APX activity can serve to directly scavenge H_2_O_2_ and maintain higher photosynthetic activity when plants are exposed to salt stress. These results suggest that the overexpression of *JctAPX* in tobacco enhanced the chloroplastic ROS scavenging system by removing H_2_O_2_, leading to increased tolerance to salt-induced oxidative stress. This study also showed that transgenic lines had significantly lower MDA contents when compared to the control lines, irrespective of whether the plants were exposed to NaCl stress or not (Figure 8B). MDA is an end product of lipid peroxidation within biomembranes, and the MDA content usually reflects the level of lipid peroxidation and indirectly reflects the extent of membrane injury [29]. These experiments indicated that enhanced APX activity resulted in reduced MDA content and conferred stress tolerance to the plants. The results of our current study are consistent with those of previous studies [30].

## 4. Experimental Section

### 4.1. Plant Materials and Treatments

Mature seeds, leaves, flowers, roots, and stems of *J. curcas* were collected in the summer from Panzhihua city, Sichuan Province, China, and immediately frozen in liquid N_2_ and stored at −70 ºC. The mature seeds were surface sterilized in 75% ethanol for 6 min and then in 0.1% HgCl_2_ for 12 min. The seeds were rinsed three times with sterile distilled water, after which their cotyledons were taken out and placed in 150 mL flasks containing 30 mL MS medium [31]. Four days later, the rooted cotyledons were transferred into pots with vermiculite-peat (1:1, *v*/*v*) medium and incubated at 28 ºC with a 16 h light/8 h dark photoperiod.

Tobacco (*Nicotiana tabacum* L.) seeds were surface sterilized and sown on plates containing 20 mL MS medium. Seeds were stratified in the dark at 4 ºC for 2 days and then transferred to a tissue culture box at 25 ºC (14 h light/10 h dark photoperiod).

### 4.2. Cloning and Sequencing of the *JctAPX* Gene

Total RNA from leaves of *J. curcas* was extracted using TRIZOL reagent (Invitrogen, Carlsbad, CA, USA) according to the manufacturer’s instructions. The first strand cDNA was synthesized using the SMART™ RACE cDNA Amplification kit (Clontech, Palo Alto, CA, USA).

The PCR product of *JctAPX* was amplified with two degenerate primers, *JctAPX*1 and *JctAPX*2 (Table 1), which were designed based on the conserved regions of the corresponding genes from other higher plants. The 5′- and 3′-ends of *JctAPX* were obtained by using the specific primers, *JctAPX*3, *JctAPX*4, *JctAPX*5, and *JctAPX*6 with a BD SMART RACE cDNA Amplification Kit (Clontech, Palo Alto, CA, USA) according to the manufacturer’s instructions (Table 1). DNA sequencing was performed by Invitrogen in Shanghai, China.

Sequences were aligned and phylogenetic trees were constructed with Clustal X (version 2.012, University of Strasbourg, Strasbourg, France) and MEGA4 software (version 4.1, Biodesign Institute, Tempe, AZ, USA).

### 4.3. Real-Time PCR

Total RNAs were extracted from different tissues of *J. curcas* or from the leaves under 400 mM NaCl stress using TRIZOL reagent (Invitrogen, Carlsbad, CA, USA) and treated with DNase (Fermentas, Burlington, ON, Canada). The reverse transcribed cDNA samples were synthesized using the OneStep RT-PCR kit (Fermentas, Burlington, ON, Canada). Expression levels of the genes were determined using the iCycler IQ Real-time PCR Detection System (Bio-Rad, Hercules, CA, USA) according to the manual for the QuantiTect SYBR Green PCR kit, and were analyzed by using the iCYcler real-time detection system (version 3.0, Bio-Rad, Hercules, CA, USA). A *JctAPX* fragment (136 bp) was amplified with the gene-specific primers *JctAPX*7 and *JctAPX* 8 (Table 1). A *J. curcas actin* gene, amplified with the primers *Actin-F* and *Actin-R* (Table 1), yielding a product of 180 bp, was used as a reference for normalizing the *JctAPX*cDNA amounts. The final relative cDNA amounts of *JctAPX* were calculated as the means of three replicates.

### 4.4. Subcellular Localization of JctAPX

To investigate the intracellular targeting of JctAPX, two DNA constructs (*p35S: GFP* and *p35S: JctAPX-GFP*) were prepared using transient expression in *Arabidopsis* mesophyll protoplasts. *GFP* was inserted into *SmaI*-*SacI* sites of pBI221 [32], yielding *p35S: GFP*. The complete coding region of *JctAPX* was subcloned into the *p35S: GFP* vector between the *BamHI* and *SmaI* sites, upstream and in frame with the GFP coding region. *Arabidopsis* mesophyll protoplasts were isolated, transfected with the above two constructs [33], and examined by confocal microscopy (Leica Microsystems, Heidelberg, Germany).

### 4.5. Plasmid Construction and *N. tabacum* Transformation

*JctAPX* was amplified from cDNA by PCR using specific primers that contain a *BamH*I site and an *EcoR* I site, and then subcloned into the cognate sites of a modified plasmid, pBI121. The *JctAPX* fragment was located between the CaMV 35S promoter and the NOS 3′ poly (A) signal to generate*p35S: JctAPX*. The construct was transformed into *Agrobacterium* (EHA105). Tobacco was transformed according to the procedure described by Lu *et al.* [34]. Transformants were selected for their ability to grow on 1/2 MS medium containing 50 μg/mL kanamycin and by PCR. T_2_ generation plants were used in all experiments unless otherwise indicated.

### 4.6. Salt Tolerance Assay

WT and transgenic tobacco seeds were surface sterilized, and sown in Petri dishes containing 1/2 MS medium. The plates were kept at 4 ºC for 2 days and then shifted to 25 ± 1 ºC. After 6 days seedings were transferred to 1/2 MS containing 200 mM NaCl.

Leaf disks were floated on 4 mL of 400 mM NaCl solution and incubated for 96 h at 24 ºC using 16 h light/8 h dark photoperiods. Leaf disks floated in sterile distilled water served as the experimental control. The chlorophyll content of the leaf disks was estimated was estimates as a measure of salt-mediated chlorophyll loss in the different transgenic plants.

### 4.7. Measurement of Chlorophyll Content

Leaf tissue (0.5 g) was homogenized in liquid nitrogen, and mixed with 7 mL cold 80% (*v*/*v*) aqueous acetone. The homogenate was filtered, and its solid residue was washed three times with 5 mL of cold 80% acetone. After finishing the pigment extraction, cold 80% acetone was added to the filtrate to a total volume of 20 mL. The chlorophyll concentration of total filtrate was measured spectrophotometrically [35]. All quantifications were performed in triplicate.

### 4.8. Determination of Lipid Peroxide

Lipid peroxidation in leaves was assayed by measuring the MDAcontent. Thiobarbituric acid (TBA)-reactive substances, representing lipid peroxidation products, were extracted by homogenization of 0.2 g of leaf in 5 mL of 0.6% (*v*/*v*) TBA solution in 10% (*v*/*v*) trichloroacetic acid (TCA). The mixture was kept in a boiling water bath for 30 min and then quickly cooled in an ice bath. After centrifugation at 13,000× *g* for 10 min, the absorbance of the supernatant was measured at 532 and 600 nm by atomic emission spectrophotometry (Analytik Jena AG, Jena, Germany). The MDA concentration was determined by its molar extinction coefficient, 155 mM^−1^ cm^−1^ [36].

### 4.9. APX Activity Assay and Quantitative Analyses of H_2_O_2_

Chloroplasts were isolated from leaves as reported previously [37]. Leaves (20 g) were cut into pieces and homogenized in extraction buffer (0.33 mM sorbitol, 50 mM HEPES/KOH (pH 8.0), 5 mM MgCl_2_). The homogenates were then filtered and centrifuged at 2000× *g* for 30 min. The pellet was suspended in phosphate-buffer saline (PBS) for measurement of chloroplast APX activity. Ascorbate (2 mM) was added to all media and solutions used in all steps, in order to ensure retention of APX activity. The APX activities were measured by monitoring the decrease in absorbance at 290 nm. The reaction was initiated by addition of 0.5 mM H_2_O_2_. One unit of APX was defined as the amount of enzyme that oxidized 1 μmol of ascorbate per min at 25 ºC [38]. Protein content was determined with a Protein Assay Kit (Bio-Rad), using bovine serum albumin as the standard.

For H_2_O_2_ content analyses, leaves (0.5 g) were homogenized with 2 mL of phosphate buffer (50 mM, pH 6.8) containing the catalase inhibitor, hydroxylamine (1 mM). The homogenate was centrifuged at 6000× *g* for 30 min. The supernatant was mixed with 1 mL of 0.1% titanium sulfate in 20% (*v*/*v*) H_2_SO_4_ and the mixture was centrifuged at 6000× *g* for 15 min. The intensity of the yellow color of the supernatant was measured at 410 nm. The H_2_O_2_ concentration was calculated using the extinction coefficient 0.28 μmol^−1^ cm^−1^ [39].

### 4.10. Data Analysis

Each treatment was carried out in triplicate, and the results are expressed as the mean ± SD. Statistical differences of data were examined by using the Student’s t-test. Each experimental value was compared with its corresponding control value. Statistical analyses were performed using SPSS 13.0 (SPSS Inc.; Chicago, IL, USA). A statistically significant difference was defined as *p* < 0.05 in all statistical analyses.

## 5. Conclusions

Our results demonstrated that overexpression of the *JctAPX* gene in transgenic plants led to improved ROS scavenging ability, one of the mechanisms that protect plants from damage caused by salt stress. This gene is a plausible candidate for improving plant tolerance to drought through genetic biotechnology approaches in the future.

## Figures and Tables

**Table 1.** Primer sequences used in the experiments.

Primer	Sequence(5′-3′)
*JctAPX1*	AG(A/T)AGGAT(G/C)AA(G/C)ATC(T/A)ACTTG(T/C)ATT
*JctAPX2*	AT(T/C)G(C/T)CAAC(A/G)ACC(T/C)AC(A/G)AGC(T/C)A
*JctAPX3*	CTTTCCAGTTGAGTATTTGGCTGCT
*JctAPX4*	CTGCAACACCATCTAGCACAATACC
*JctAPX5*	TTCTTTTTGATGATCCTTCGTTCAG
*JctAPX6*	ATACTCAACTGGAAAGAGAGAATTG
*JctAPX7*	GAAACTCCTTCAGCCAATCAA
*JctAPX8*	TGAAACATCCACCCTTCCATAC
*Actin-F*	ATGAGCTTCGAGTTGCACCA
*Actin-R*	AGCATCAGTGAGATCACGAC
